# A Licorice-Flavored Edema: A Case Report of Glycyrrhizic Acid Toxicity From Chronic Licorice Root Consumption

**DOI:** 10.7759/cureus.34425

**Published:** 2023-01-31

**Authors:** Jean-Samuel Blanpain

**Affiliations:** 1 Emergency Medicine, Centre Hospitalier Régional Sambre et Meuse (CHRSM), Namur, BEL

**Keywords:** licorice, palpebral edema, lower limb edema, glycyrrhizin, pseudohyperaldosteronism

## Abstract

This article presents a case study of a 49-year-old patient who was admitted to the emergency department with hypertension, edema, and intense fatigue caused by excessive consumption for three weeks of licorice herbal teas purchased on the internet. The patient was only taking antiaging hormonal treatment. The examination revealed bilateral edema of the face and lower limbs, and blood tests showed discrete hypokalemia (3.1 mmol/L) and low aldosterone levels. The patient revealed that she had been consuming large amounts of licorice herbal teas to compensate for the lack of sweetness in her low-sugar diet. This case study highlights that although licorice is widely used for its sweet taste and has medicinal properties, it can also have a mineralocorticoid-like activity that can lead to apparent mineralocorticoid excess (AME) when consumed in excess. The main component of licorice responsible for these symptoms is glycyrrhizic acid, which increases the availability of cortisol by decreasing its catabolism and has a mineralocorticoid effect through the inhibition of the enzyme 11-β-hydroxysteroid dehydrogenase (11-β-HSD) type 2. The case also discusses the clinical effects of licorice consumption, such as sodium retention and potassium excretion, leading to potential cardiovascular complications, as well as a differential diagnosis of similar clinical presentations mainly based on laboratory findings including aldosterone level and plasma renin activity (PRA). The potential dangers of consuming excessive amounts of licorice are well established, and we advocate stricter regulations and increased awareness and education for both the general public and the medical profession about these negative side effects and suggest that physicians should consider licorice consumption in their approach to patients’ lifestyles and diets.

## Introduction

Derived from *Glycyrrhiza glabra*, a well-known plant since antiquity, with many medicinal properties claimed (such as anti-infective properties and abdominal pain or cough relief), licorice is widely used nowadays by the food industry for its sweet taste [1]. Unfortunately, this broad use hides from the population the fact that this substance exerts an intrinsic and indirect mineralocorticoid activity and can lead to apparent mineralocorticoid excess (AME) if consumption exceeds the suggested upper limit. We report here the case of a patient who developed hypertension and edema induced by the consumption of licorice herbal teas purchased on the internet.

## Case presentation

A 49-year-old patient was admitted to the emergency department for edema (initially periorbital and then extended to the lower limbs and the face) and intense fatigue for four days. She noticed that she had gained 3 kg since her last weighing four days ago, as well as higher blood pressure (BP) than usual (BP: 140/90 mmHg) (the usual value is around 120/70 mmHg). She denied any other complaints. Her past medical history was only significant for controlled migraine attacks years ago. She does not smoke or drink alcohol. Followed for three years in a private antiaging clinic, her usual treatment consisted of pregnenolone, dydrogesterone, dehydroepiandrosterone (DHEA), and lyophilized thyroid extracts. There had been no change in dosage for more than a year.

In the emergency department, her vital signs were as follows: symmetrical blood pressure of 141/92 mmHg, pulse of 67 beats per minute, 99% ambient air saturation, breathing rate of 18 breaths per minute, and a measured temperature of 37.1°C.

We observed bilateral edema of the face and lower limbs, with a positive bilateral Godet sign. The cardiopulmonary examination did not reveal any abnormalities, and the abdominal and neurological examinations were also unremarkable.

Her laboratory results showed the absence of inflammatory syndrome, preserved renal function, mild hypokalemia measured at 3.1 mmol/L (normal range: 3.5-5 mmol/L) without any other abnormality to the serum ionogram, especially without metabolic alkalosis, thyroid balance in normal values, pro-B-type natriuretic peptide (pro-BNP) measured at 855 ng/L (normal range: 0-146 ng/L) and D-dimers at 0.82 mg/L (normal range: 0-0.5 mg/L), serum protein in normal values (in particular, the albumin level was 41.2 g/L (normal range: 40.2-47.6 g/L)), kaliuria measured at 34 mmol/L, and natriuria at 79 mmol/L, without detected proteinuria. An EKG (without significant abnormalities) and a chest X-ray (signs of slight hilar vascular overload without cardiomegaly) completed the initial assessment.

The next morning, a transthoracic cardiac ultrasound (no sign of left or right heart failure, but discrete pericardial effusion) and an ultrasound of the lower limbs (no thrombotic phenomenon observed) were performed. The initial biology was supplemented by a renin and aldosterone study, with an aldosterone level < 1 ng/dL (normal range: 2.5-39.2 ng/dL). A dexamethasone suppression test was programmed to rule out Cushing’s disease and turned out to be negative. The patient was then offered to continue the assessment as an outpatient with oral potassium supplementation.

However, she was admitted again to the emergency department after four days. Edema increased, as well as the blood pressure measured at home (around 160/95 mmHg). The control laboratory did not find any new abnormalities. On the other hand, the patient told us for the first time that in parallel with a low-sugar diet recently initiated, she had been compensating for the lack of sweet taste for three weeks by a 4-5 times a day consumption of infusion based on dried licorice root bought on the internet (around 10 g per infusion). Regarding the lowered aldosterone level highlighted at prior admission, it was decided to discontinue licorice consumption.

Without further modification (and maintaining her antiaging hormonal treatment), the patient observed over the next 15 days a complete improvement of edema and normalization of blood pressure at home. At three months, the patient did not report any recurrence.

## Discussion

As a reminder, aldosterone is the most powerful adrenal mineralocorticoid, causing sodium retention and potassium excretion. The secretion of aldosterone is primarily modulated by the renin-angiotensin system, the reduction of blood volume and flow at the level of the afferent renal arterioles, or hyponatremia resulting in the secretion of renin [2].

The diagnosis of hyperaldosteronism is suggested by the association of high blood pressure and hypokalemia (which may be severe and lead to an elongation of the QT and/or ventricular arrhythmia), with hypernatremia in some cases. Sodium retention can lead to generalized edema or, more severe, cardiac decompensation and pulmonary edema [3].

The clinical picture can also be supplemented by asthenia, paresthesia, transient paralysis, or even tetany. Symptoms of apparent mineralocorticoid excess (AME) are similar to those of primary hyperaldosteronism [4].

In the case of our patient, we observed a pattern predominated by extensive edema associated with recent-onset hypertension and intense fatigue, but without profound hypokalemia. A line of thought about the absence of severe hypokalemia could be the antiaging hormonal treatment taken by our patient. Although it is challenging to confirm their influence on the clinical picture, progesterone compounds such as dydrogesterone can have antimineralocorticoid, antiandrogen, and pro-glucocorticoid effects, and it could effect to kalemia. Furthermore, the empiric use of thyroid extract without a clear diagnosis of hypothyroidism should also be discouraged.

During the initial biological assessment of suspicion of hyperaldosteronism, an aldosterone and plasma renin activity (PRA) study should be performed. The latter is usually measured four weeks after intravenous injection of radioactive isotopes, ideally excluding any medication interfering with the renin-angiotensin system (e.g., beta-blockers, thiazide diuretics, angiotensin II receptor blockers, and angiotensin-converting enzyme (ACE) inhibitors) [2]. Unfortunately, as our patient improved quickly after licorice intake cessation, she did not present for the test. However, since our patient’s aldosterone level was low, this was unnecessary for differential diagnosis.

Indeed, high plasma aldosterone levels, often greater than 15 ng/dL (>0.42 nmol/L), and low plasma renin activity suggest primary hyperaldosteronism. On the other hand, high levels of both plasma renin activity and aldosterone should evoke secondary hyperaldosteronism (due to the excessive stimulation of a functional renin-angiotensin-aldosterone system, e.g., renal hypoperfusion). However, in the presence of low levels of plasma renin activity (PRA) and aldosterone, an apparent mineralocorticoid excess (AME) is suspected, i.e., excess of mineralocorticoids other than aldosterone (e.g., licorice ingestion and Cushing’s or Liddle’s syndrome) [2].

Licorice is derived from the plant *Glycyrrhiza glabra*. Its name is taken from the ancient Greek “glycos,” meaning sweet, and “rhiza,” meaning root [3]. This plant has been known and used since antiquity for a wide variety of uses, some for claimed medicinal purposes (such as anti-infective properties and abdominal pain or cough relief) and others for its sweet taste, derived from glycyrrhizic acid (of which sweet taste is 50 times stronger than sugar) [1,5]. It is this role of substitute for sugar that prompted our patient to consume licorice many times a day when starting a low-sugar diet. Unfortunately, most people are unaware of the negative consequences of repeated consumption of licorice and consider this food an excellent dietary alternative. Moreover, its use is free and is not subject to strict supervision despite many proven side effects [3]. Some societies have made recommendations on the daily intake limit. For example, the European Union recommends that regular intake of glycyrrhizin should not exceed 100 mg per day (found in 60-70 g of licorice) [6]. Our patient was consuming licorice root infusions 4-5 times a day, with approximately 10 g per serving, totaling at least 50 g per day. The European Commission has also established a directive on the labeling of certain food products containing glycyrrhizin and its ammonium salt [1].

The main mode of action of glycyrrhizin is through its active metabolite, glycyrrhizic acid, which inhibits the enzyme 11-β-hydroxysteroid dehydrogenase (11-β-HSD) type 2, leading to increased cortisol availability. Although the affinity of cortisol and aldosterone for the mineralocorticoid receptor is about equal, 11-β-HSD type 2 inhibition results in a cortisol-induced mineralocorticoid effect as cortisol is much higher in concentration compared to aldosterone and successfully competes for the receptor [7]. The aldosterone-like effect of licorice is also explained by a direct action on mineralocorticoid receptors, but this mechanism is weak compared to the first [3].

Faced with a picture suggestive of AME, even more in the absence of potentially incriminated medication, it is necessary to undertake a thorough dietetic history. If licorice consumption is retained, the diagnosis can be confirmed by stopping any licorice consumption and observing a slow improvement thereafter [3]. If in doubt, licorice abuse can be confirmed by a urine assay of glycyrrhizic acid using high-performance liquid chromatography coupled with a mass spectrometer [8].

Licorice-induced AME is reversible, taking into account that the inhibitory action on 11-β-HSD type 2 lasts about two weeks. In addition, the renin-angiotensin-aldosterone system may take up to six months to recover to its previous level, which may be temporarily supplemented with potassium-saving diuretics, spironolactone, and dexamethasone, keeping in mind that glucocorticoids may be too strong and have more side effects [3,9].

## Conclusions

This clinical case should remind us that pharmaco-active substances are sold over the counter and must be unmasked by careful anamnesis. Emphasis should be placed on stricter legislation and information for both the general public and the medical profession. Indeed, because of its many negative side effects, especially cardiovascular, physicians should consider the consumption of licorice in their lifestyle and dietary approach.
